# Supplementary material for: Laryngopharyngeal reflux, gastroesophageal reflux and dental disorders: A systematic review
Source: PLoS One. 2020 Aug 14;15(8):e0237581. doi: 10.1371/journal.pone.0237581 (PMC7428125; doi:10.1371/journal.pone.0237581)
Supplement: Attachment — Submitted filename: Response to Reviewers.docx [file pone.0237581.s002.docx]

PLOS ONE

Mons, July, 2020

Dear Professor Cammaroto,

I’m sending the revised Systematic Review entitled: “**Laryngopharyngeal Reflux, Gastroesophageal Reflux and Dental Disorders: A Systematic Review***.*" (by Lechien et al.).

I undersigned, Jérôme R. Lechien, certify on behalf of all co-authors that the work has not been published previously or submitted elsewhere for review. Any author has financial interest in the subject under discussion. All authors have read and approved the manuscript. Would you be so kind to consider the present manuscript and send us the Reviewer’s comments.

We considered the comments of the reviewer and the editor.

Reviewer #1: This is an interesting review regarding laryngopharyngeal reflux, gastroesophageal reflux and dental disorders. No reviews about this specific topic have been published so far. Besides, the study is well conducted and all aspects concerning the topic are analyzed.
Thank you.

Recently, in patients with laryngopharyngeal reflux pepsin has also been identified in tears, please mention this possibility.
We modified the paragraph in the introduction: introduction, line 6 : « *Thus, pepsin has been identified in the laryngeal [5], hypopharyngeal [6], oral [7], nasal [7], tears [8], and Eustachian mucosa as well as in the secretion of chronic rhinosinusites^9^ and chronic media otitis [10].* »

Is there any evidence regarding reflux treatment and dental conditions? If they exist, they should be mentioned.

There is one study, but the data of this study (Wilder-Smith et a., 2017) overlapped those of another study (Wilder-Smith, 2015).

We included these findings in the discussion : last paragraph of the discussion : « *Note that, to date, only one study investigated the dental health of GERD patients throughout a PPI clinical course. Wilder-Smith et al. found that erosive tooth wear did not progress over the 12-month PPI course [66].* »

Best regards,

Jérôme R. LECHIEN, M.D., Ph.D., M.S.

Head and Neck surgery, Laboratory of Anatomy and Cell Biology, Faculty of Medicine, University of Mons (UMONS), Avenue du Champ de mars, 6, B7000 Mons, Belgium, [Jerome.Lechien@umons.ac.be](mailto:Jerome.Lechien@umons.ac.be)

Telephone: +32 65 37 35 84

Fax: +32 65 37 31 42
